# Associations Between Vocal Arousal and Dyadic Coping During Couple Interactions After a Stress Induction

**DOI:** 10.1007/s41042-023-00087-5

**Published:** 2023-03-17

**Authors:** Lisanne J. Bulling, Peter Hilpert, Isabella C. Bertschi, Ana Ivic, Guy Bodenmann

**Affiliations:** 1 Department of Psychology, Clinical Psychology for Children/Adolescents & Couples/Families, University of Zurich, Binzmühlestrasse 14/23, Zürich, CH – 8050 Switzerland; 2 Department of Psychology, Université de Lausanne, Lausanne, Switzerland; 3 Family Translational Research Group, New York University, New York, United States

**Keywords:** Fundamental frequency, Dyadic coping, Emotional arousal, Voice stress, Emotional resonance, Social support

## Abstract

It is well known that although relationship external stressors can harm couples, dyadic coping behavior can buffer the negative effects of stress. Thus far, however, less is known about how vocally encoded stress (i.e., *f*_0_) might affect the stress-coping process in couples during an interaction. Therefore, the goal of the current study was to compare two different stress hypotheses (i.e., paraverbal communication stress hypothesis and emotional resonance hypothesis). We observed 187 mixed-gender couples (*N* = 374 participants) interacting naturally after an experimental stress induction (Trier Social Stress Test), for which couples were randomly allocated into three groups (women stressed, men stressed, and both stressed). Results of a multi-group actor-partner interdependence mediation model (APIMeM) show that either the paraverbal communication stress hypothesis or the emotional resonance hypothesis could be confirmed, depending on whether the man, the woman, or both partners were stressed.

## Associations Between Vocal Arousal and Dyadic Coping During Couple Interactions After a Stress Induction

Romantic relationships are defined by the interactions between romantic partners (Rogers & Escudero, 2014). A frequent type of interaction between romantic partners is conversations in which they share details of recent stressful experiences with one another (Bodenmann, 2005). The couple’s attempt to cope with these stressful experiences together, or dyadic coping (Bodenmann, 1995a), has been consistently linked to well-being in romantic relationships (for a meta-analysis see Falconier et al., 2015). However, less is known about the factors that facilitate the dyadic coping process. This process is mainly based on three factors: (i) that Partner A, who is stressed, seeks support by communicating their stress in a way that Partner B can properly decode and understand, (ii) that Partner B empathizes with Partner A’s experience of the stressful event and provides problem-focused and emotion-focused support, and (iii) that the Partner A is finally satisfied with Partner B’s support (match between need and support provision). The goal of the current study is to examine the causal relationship between those factors.

### Stress and Dyadic Coping in Romantic Relationships

Romantic relationships can be negatively impacted by the stress that partners encounter outside of their relationship. When a person’s stress level exceeds their resources, it can spill over into the relationship and affect both partners (for a review see Randall & Bodenmann 2009). For example, compared to people with fewer everyday stressors, people with more everyday stressors report lower levels of relationship satisfaction (e.g., Bodenmann et al., 2007; Falconier, Nussbeck, et al., 2015), and a larger number of conflicts (Bolger et al., 1989). People with more everyday stressors also have a higher risk for divorce (Bodenmann & Cina, 2006). These harmful effects of stress can be mitigated by couples through dyadic coping as described in the Systematic-Transactional Model (Bodenmann, 1995a, 2005).

The Systematic-Transactional Model (STM; Bodenmann, 1995, 2005) seeks to explain why stress affects couples differently. Simply put, the model’s three main components—how a stressed person communicates stress, how the partner responds to the stress communication by providing support, and how the provided support might help the stressed person—are meant to predict if a couple copes with stress successfully or becomes negatively affected by it. The dyadic coping process consists of a transactional sequence that starts with Partner A’s stress communication (i.e., support seeker). This stress communication can be verbal (e.g., verbal description of the event and the emotions associated with it), non-verbal (e.g., nervous body movements, gestures, facial expressions), and paraverbal (e.g., changes in the tone of voice; Baucom et al., 2011; Bodenmann, 1995; Fischer et al., 2015). For example, a person who went through a difficult job interview may express their emotional stress by describing the interview verbally in a neutral, matter-of-fact way to their partner while, at the same time, expressing their emotional distress paraverbally through a high-pitched voice. These stress signals, each by itself and in their combination, are important because it is only through an unambiguous communication of the stress experience that partners can accurately understand one another’s stress and respond to it in a manner that matches the other’s needs (Bodenmann, 1995, 2005). An adequate stress communication that allows the partners to understand one another’s stress is therefore the first step in a successful dyadic coping process.

The second step in a successful dyadic coping process is that Partner B (i.e., support provider) perceives the stress communication and responds with supportive reactions instead of failing to respond or reacting with their own stress communication (Bodenmann, 1995, 2005). A supportive reaction to a partner includes behaviors such as showing interest through attentive listening, emphasizing, understanding, showing solidarity, and providing encouragement. These coping behaviors have been linked to relationship satisfaction in both the general population (Bodenmann & Cina, 2006; Herzberg, 2013) and in couples going through stressful life periods (Molgora et al., 2019). The third step in a successful dyadic coping process is Partner A’s satisfaction with Partner B’s support provision. Thus far, however, little is known about this three-step process, as most studies so far relied on self-reports to assess stress communication, support provision, and satisfaction with the support provision (e.g., DCI; Gmelch et al., 2008).

### The Emotional Climate of Dyadic Coping

Dyadic coping is a form of emotional co-regulation (Randall et al., 2021). Accordingly, emotional arousal is an integral component of the dyadic coping process in which both partners should experience some degree of emotional arousal. The STM stresses the importance of gaining an *emotional* understanding of the partner’s stress (Bodenmann, 1995, 2005); knowing only the situational and factual aspects of the partner’s stress is not enough to adequately provide dyadic coping that matches the needs of the stressed partner, one must also understand the *emotions* elicited by the stressful experience (Bodenmann & Randall, 2012). An adequate stress communication that allows the partners to understand one another’s emotional experience is therefore a requirement for a successful dyadic coping process (Bodenmann et al., 2016).

One way for the partner to perceive the emotions of the support-seeking partner is through the support-seeking partner’s stress communication. This stress communication can be paraverbal as well as verbal or nonverbal. Characteristics of the voice contain large amounts of information about the speaker’s emotional experiences (Juslin & Scherer, 2008). The most perceptually salient characteristic of the voice is its fundamental frequency (*f*_0_). The *f*_0_ of the voice reflects the rate of vocal cord vibration and corresponds to what listeners perceive as voice pitch (Hart et al., 1990). Increases in *f*_0_ can be triggered by socially stressful experiences and can be simulated in the laboratory with stress induction protocols (Pisanski et al., 2018) and physiological stress signals (Pisanski & Sorokowski, 2021). Yet, unlike physiological stress signals (e.g., an increase in heart rate or skin conductance), vocally encoded emotional arousal can be perceived directly by the listener. Listeners can infer a speaker’s emotions from their voice with relatively high accuracy, with a higher *f*_0_ mean indicating highly aroused positive or negative emotions, such as anger or happiness (Laukka et al., 2005). Therefore, a speaker’s paraverbal stress communication, which can be measured as an increase in *f*_0_, should help the partner perceive and respond to the speaker’s emotions. In the last decade, studies found that *f*_*0*_ is associated with a demand/withdraw pattern (Baucom et al., 2011), poorer long-term memory of communication skills (Baucom et al., 2012), relationship satisfaction (Fischer et al., 2019), and support (Fischer et al., 2015).

### Emotional Resonance

Another way for the partner to understand the emotions of the support-seeking partner is through emotional resonance; that is, the partner feels a similar level of emotional arousal as the support-seeking partner. This emotional resonance or empathic resonance of stress has recently begun to be more closely investigated (for a review see Engert et al., 2019). During the Trier Social Stress Test (Kirschbaum et al., 1993), a standardized and well-validated social stress induction that includes a mock job interview and a mental arithmetic test, experimenters tasked with inducing stress in study participants showed increases in cortisol release that was proportional to the cortisol release of the participants (Buchanan et al., 2012). In another study using the TSST, emotional resonance was found to be stronger between romantic partners than between strangers (Engert et al., 2014).

Emotional resonance can be viewed as an aspect of empathy that entails the affective reaction to another person’s emotions (i.e., feeling the emotions of another person) which has been distinguished from cognitive empathy (i.e., understanding the emotions of another person; (for a review see Cuff et al., 2016). Empathy is associated with better dyadic coping (Leuchtmann et al., 2018; Levesque et al., 2014; Verhofstadt et al., 2008, 2016) and higher relationship satisfaction (Kimmes et al., 2014; Ulloa et al., 2017). Thus, during conversations about stressful experiences, higher emotional arousal of the support-providing partner can be viewed as a sign of emotional resonance which, in turn, should help the support-providing partner offer support that matches the emotional needs of the support-seeking partner.

### Providing Support while Stressed

The STM distinguishes between the partner who communicates stress and the responding partner. The standard procedure for prompting a dyadic coping conversation in a behavioral observation study is to ask couples to have two conversations: One about a relationship-external stressor affecting one partner and one about a relationship-external stressor affecting the other partner. However, in couples’ natural interactions the role of the stress-seeking and the support-providing partner may be less clear. What happens when both partners come home from work stressed? Such scenarios are likely to occur regularly, leading to a competing need for support and requiring both partners to switch between seeking and providing support.

Being responsive to a partner’s needs may be difficult at times when support providers are faced with their own stressors. Daily stress may interfere with individuals’ capacity to notice a partner’s support needs. For example, acute stress can interfere with the ability to recognize a speaker’s emotions (Israelashvili et al., 2020). Daily stress may also interfere with individuals’ capacity to enact support. For instance, even when men noticed that their partner was seeking support, they were less likely to provide it on days on which they were coping with their own stress (Neff et al., 2021).

### The Current Study

An experimental approach was used to study how the complex process of stress and dyadic coping unfolds during couple interactions. Mixed-gender couples were invited into the laboratory, where either the men, the women, or both underwent a stress induction. Directly after the stress induction, the stress-coping process was observed during a natural interaction period of 8 min. The three factors of the stress-coping process were measured in the following way: (i) para-verbal stress expressions were assessed by each partner’s mean *f*_0_; (ii) each partner’s dyadic coping behavior was coded following the System to Evaluate Dyadic Coping (SEDC; Bodenmann 1995b); (iii) and after the interaction, participants were asked to rate how satisfied they were with their partner’s coping behavior during the interaction.

We propose two hypotheses, a stress communication, and an emotional resonance hypothesis. First, the *Paraverbal Stress Communication Hypothesis* is based on STM and suggests that the *stressed* partner’s emotional arousal is positively associated with how much dyadic coping Partner B provides, which in turn increases the satisfaction level of the stressed partner at the end of the interaction (H1). This should be the case when either the women (Group 1) or the men are stressed (Group 2). However, the outcome is unclear when both partners are stressed as they are at the same time support seekers and support providers.

Second, the *Emotional Resonance Hypothesis* assumes that higher emotional arousal in the *unstressed* partner is a proxy for empathy. Feeling empathic towards the stressed partner helps to provide adequate support which in turn leads to the stressed partner being more satisfied with the support provided by the unstressed partner (H2). Thus far, one has to assume that the stress-coping process will be disturbed with the roles of support seeker and support provider become unclear when both partners are stressed.

The established approach in psychological scientific publications is that one should not make causal statements based on observational data (i.e., correlation is not causation), yet new publications (Rohrer, 2018; Rohrer et al., 2021) suggest that this hinders progress in psychology and it would be much better to make implicit causal assumptions explicit (Grosz et al., 2020; Rohrer, 2018; Rohrer et al., 2021). This suggestion is not just based on research about causal inference over the last decades (Dawid, 1979; Pearl, 1993, 2009; Peters et al., 2017; Rubin, 1974) but has started to be accepted in psychology (Bullock et al., 2010; Grosz et al., 2020; Rohrer, 2018; Rohrer et al., 2021). In contrast to the rather problematic approach in psychology that results from a causal model (i.e., regression models) can only be interpreted as an association, the new approach allows interpreting the results as causal under the assumption that the causal model corresponds to the Data Generating Process (DGP), i.e., that the model reflects the underlying causal process in the real world (Rohrer, 2018), acknowledging that testing this assumption is difficult.

**Fig. 1:**
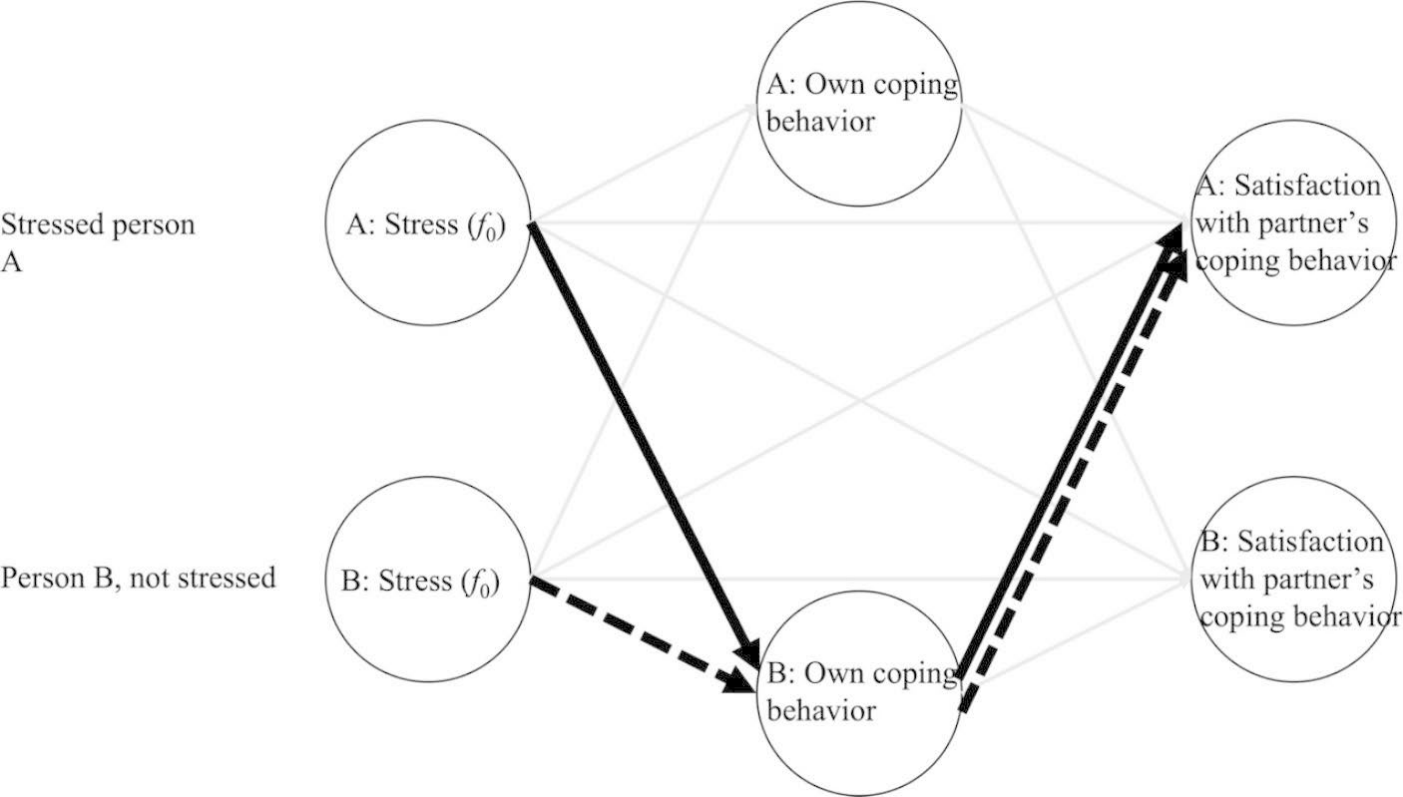
* A Graphical Representation (i.e., DAG) of The Causal Relationships, Specifically The Paraverbal Stress Communication Hypothesis (in bold line) And The Emotional Resonance Hypothesis (bold dashed line)* *Note*. A directed acyclic graph illustrating the causal flow of the paraverbal stress communication hypothesis and the emotional resonance hypothesis. Based on theoretical assumptions, the paraverbal stress communication hypothesis predicts that Person A’s voice stress (i.e., the stressed person) affects the coping behavior of person B, which in turn influences how satisfied Person A is the provided support (bold lines). In contrast, the emotional resonance hypothesis predicts that Person B (i.e., the non-stressed partner) is positively infected by B’s stress, which then influences B’s own coping behavior, which in turn influences partner A’s satisfaction with person B’s coping behavior (dotted lines).

To make our causal assumptions unambiguous, we are using a causal graph, i.e., a directed acyclic graph (DAG; Fig. 1; Pearl 2009). The paraverbal stress communication hypothesis (H1) is represented by a solid line in Fig. 1, whereas the emotional resonance hypothesis (H2) is depicted by a dashed line. Although both hypotheses might be interpreted as a mediation, this is not suggested by our theoretical assumptions. Rather, the causal link is a chain—Partner A or B’s *f*_0_ affects Partner B’s coping behavior, and Partner B’s coping behavior affects Partner A’s satisfaction with the provided coping.

## Method

### Participants

Couples residing in BLINDED were recruited through advertisements in newspapers, magazines, and websites. Inclusion criteria for the study were a minimum relationship length of 12 months, age 20 to 45, and fluency in BLINDED. Because the larger study protocol included cortisol measurements, women were also required to have a regular menstrual cycle and to participate during the luteal phase of their cycle. Participants were excluded from the study if they smoked more than 10 cigarettes per day, had a chronic illness, took medication, were pregnant or breastfeeding, and have participated in the TSST before. Out of the 867 couples who responded to the advertisements, 277 did not meet inclusion criteria, 152 declined because of scheduling conflicts, and 240 declined after learning more about the study. The result is 198 mixed-gender couples that participated in the study in 2008 and 2009. The 198 couples were randomly assigned to one of three experimental groups: Group 1, where only the woman in each couple participated in the TSST; Group 2, where only the man in each couple participated in the TSST; and Group 3, where both partners participated independently and simultaneously in the TSST. Seven couples were excluded because of missing video data. The final sample consisted of 187 couples (64 couples in Group 1, 63 couples in Group 2, and 60 couples in Group 3). The average age for women is 26.4 years (*SD* = 5.7) and 28.5 years (*SD* = 6.3) for men. Many were in college (women: 56%; men: 40%) and the average relationship length was 4.2 years (*SD* = 3.7); 17% of all couples were married, and 13% were raising children together^1^.

### Procedure

Upon arrival at the laboratory, couples were informed about the experiment and provided consent. Each partner completed the first set of questionnaires. Couples were then left alone in a waiting room for eight minutes while their interaction was recorded with a camera. Couples received no instructions other than to remain seated thus allowing for a natural interaction to unfold. After eight minutes, either the female (Group 1), the male (Group 2), or both partners separately and simultaneously (Group 3) participated in the TSST (Kirschbaum et al., 1993). In Groups 1 and 2, the partner who did not participate in the TSST remained in the waiting room; in Group 3, both partners participated in the TSST simultaneously in separate rooms. After the TSST, participants were accompanied back to the waiting room where the couple waited together for another eight minutes while being video recorded. After the second waiting period, each partner completed the second set of questionnaires before the couples were debriefed about the goals of the study, informed about the video recordings in the waiting room, and paid 100 Blinded (~ U.S. $100) for their participation. The experiment lasted about 2.5 hours in total. The study protocol was approved by the Institutional Review Board of the University of Zurich. The study „The impact of external stress on couples’ interaction” (Bodenmann, Heinrichs, & Bradbury) was funded by the Swiss National Science Foundation (SNF 100,014–115,948, SNF 100,014–129,627 and P2ZHP1_164959).

### Measures

***Fundamental frequency.*** Audio data was recorded with the microphone from a camcorder. Afterward, audio files were manually segmented (excluded cross-talk, laughter, and noise) and saved to separate files for each partner. *f*_0_ was estimated in 0.5-second intervals from the person’s audio segments during the 8-minute conversation using Praat (Boersma, 2001) with a bandpass filter of 75 to 200 Hz for men and 150 to 350 Hz for women. Cross-talk, laughter, and noise were removed.

***Observed dyadic coping behavior***. The interaction videos were rated in 10-second sequences according to the SEDC (Bodenmann, 1995b). Positive dyadic coping behaviors were rated on seven subscales individually for each partner (e.g., problem-focused, emotion-focused, interested listening, non-verbal, nonverbal support, asking questions, asking questions with physical contact, verbal emotional support, verbal emotional support with body contact) and a sum score was computed across all subscales and sequences. All videos were rated by two coders. These coders were intensively trained beforehand. A total of 10% of the videos were rated by both coders, indicating a good interrater reliability (Cohen’s k = 0.87). For the remaining 90% of the videos, one coder rated women’s behavior and the other men’s behavior.

***Perceived dyadic coping.*** Perceived dyadic coping was measured with a state version of the dyadic coping subscale (Gmelch et al., 2008). Participants were prompted to evaluate their partner’s dyadic coping behavior during the interaction that took place after one or both partners had participated in the TSST on three items: “I felt emotionally supported by my partner.”, “In conversation with my partner, I felt in good hands and at ease.”; “My partner made me feel that he understood me.” Items were rated on a five-point Likert-type scale ranging from 1 (*not at all*) to 5 (*very much*). In the current study, internal consistencies for men (α = 0.87) and women (α = 0.82) were acceptable.

### Statistical Analyses

The main goal of the current study was to test the (1) paraverbal stress communication hypothesis and (2) emotional resonance hypotheses. We used a specific structural equation modeling approach which enable us to deal with the interdependent data—a multi-group Actor-Partner Interdependence Mediation Model (APIMeM; Ledermann et al., 2011). This APIMeM allows us to test both hypotheses simultaneously (see Fig. 1). First, a fully saturated model was computed. In a stepwise procedure, we tested if the hypothesized effects were the same when only women were stressed (Group 1) compared to when only men were stressed (group 2) by constraining those paths to be equal, using a chi-square difference test and also evaluated the model fit of the constrained model. A constraint across two paths was only kept when the chi-square difference test was not significant and the fit indices for the constrained model was indicating good model to data fit (Hu & Bentler, 1999).

We used R version 4.1.2 (*The R Project*, 2015), the psych package for descriptive statistics (Revelle, 2022), and the lavaan package (Rosseel, 2012) for the APIMeM. The standard errors were bootstrapped based on 1000 samples. Data and syntax can be accessed here: https://osf.io/ze6w8/files/osfstorage.

## Results

### Preliminary Analyses

Means and standard deviations for all study variables for women and men in the three experimental groups are shown in Table 1. First, we tested whether the randomization was successful in relation to participants’ age. Analyzing the study variables, the non-stressed partners provided significantly more dyadic coping behavior in comparison to the stressed partner in Group 1 and 2, which also significantly differed from the dyadic coping behavior observed when both partners were stressed.

**Table 1:** *Differences in the Age, Fundamental Frequency, Dyadic Coping Behavior, and Satisfaction with Perceived Dyadic Coping Between the Three Experimental Groups*

Variable	Group 1 (women stressed) *M(SD)*	Group 2 (men stressed) *M(SD)*	Group 3 (both stressed) *M(SD)*	*p*
Age__women_	25.9(5.3)	26.0(5.8)	26.4(5.8)	0.609
Age__men_	28.2(6.3)	28.1(6.2)	28.0(6.0)	0.853
Rel.duration	4.3(3.7)	4.6(3.7)	3.8(3.9)	0.438
*f*_0_women_ (Hz)	223.5(22.2)	229.4(20.9)	226.7(16.7)	0.268
*f*_0_men_ (Hz)	123.9(13.0)	122.7(10.8)	123.3(14.5)	0.869
DC__women_	1.2(1.6)_ab_	18.0(8.5)_ac_	11.0(5.7)_bc_	< 0.001
DC__men_	20.5(9.2)_de_	1.2(1.9)_df_	12.4(7.3)_ef_	< 0.001
Sat.DC__women_	4.3(0.7)	4.2(0.7)	4.3(0.8)	0.430
Sat.DC__men_	3.9(0.8)_g_	4.2(0.9)	4.4(0.7)_g_	0.004

*Note.* M = mean; SD = standard deviation. Two-tailed p-values are based on one-way ANOVAs. Subscripts (e.g., _a_) indicate significant differences between two groups. *f*_0_ = fundamental frequency; DC = coded dyadic coping behavior; Sat.DC = satisfaction with partner’s dyadic coping; Hz = Hertz.

Bivariate correlations between study variables are presented in Table 2. Across all groups, results show that women’s coded coping behavior is associated with men’s satisfaction with the provided support and vice versa.

**Table 2:** *Intercorrelations Among All Study Variables*

	f_0_w_	f_0_m_	DC__w_	DC__m_	Sat.DC__w_
f_0_m_	0.16				
DC__w_	0.13	0.02			
DC__m_	− 0.10	0.11	**− 0.61**		
Sat.DC__w_	0.17	0.11	0.02	**0.18**	
Sat.DC__m_	**0.17**	0.13	**0.23**	− 0.10	**0.38**

*Note*. w = women; m = men; *f*_0_ = fundamental frequency; DC = coded dyadic coping behavor; Sat.DC = satisfaction with partner’s dyadic coping. All significant correlations are in bold (*p* < .05; two tailed).

### Multi-group APIMeM

We relied on common fit indices to evaluate to model to data fit. Acceptable model fit is indicated by a CFI above 0.95, a RMSEA smaller than 0.08, and a SRMR smaller than 0.08 (McDonald & Ho, 2002). Overall, the results show good model to data fit: χ2(*df* = 45) = 125.8, *p* < .001, CFI = 1.00, RMSEA = 0.000, RMSEA = 0.014. All the results of the multi-group APIMeM can be found in Table 3.

**Table 3:** *APIMeM Analyses Relating Emotional Arousal (f0), Coded Dyadic Coping Behavior, and Satisfaction with Partner’s Dyadic Coping Behavior*

			Group 1 (women stressed)				Group 2 (men stressed)				Group 3 (both stressed)		
			β	*SE*	*p*		β	*SE*	*p*		β	*SE*	*p*
Direct Effects													
Actor effects													
*f*_0w_	→ DC_w_		0.018	0.008	0.879		− 0.097	0.045	0.380		**0.328**	0.051	0.030
*f*_0w_	→ Sat.DC_w_		**0.230**	0.004	0.031		**0.391**	0.006	0.021		− 0.122	0.008	0.494
DC_w_	→ Sat.DC_w_		0.025	0.053	0.824		0.093	0.011	0.435		**0.318**	0.020	0.031
*f*_0m_	→ DC_m_		**0.251**	0.069	0.010		− 0.026	0.030	0.880		0.059	0.093	0.750
*f*_0m_	→ Sat.DC_m_		**0.120**	0.004	0.031		0.020	0.011	0.880		0.130	0.007	0.332
DC_m_	→ Sat.DC_m_		− 0.126	0.013	0.371		0.084	0.047	0.396		0.162	0.013	0.217
Partner effect													
*f*_0w_	→ DC_m_		− 0.127	0.053	0.273		0.210	0.013	0.150		0.073	0.069	0.645
*f*_0w_	→ Sat.DC_m_		0.072	0.005	− 0.592		**0.315**	0.005	0.007		− 0.155	0.006	0.239
DC_w_	→ Sat.DC_m_		− 0.086	0.060	0.445		**0.258**	0.012	0.025		**0.217**	0.012	0.025
*f*_0m_	→ DC_w_		− 0.097	0.015	0.447		**0.228**	0.069	0.010		− 0.095	0.049	0.448
*f*_0m_	→ Sat.DC_w_		**0.262**	0.006	0.019		0.024	0.009	0.856		− 0.104	0.008	0.494
DC_m_	→ Sat.DC_w_		**0.330**	0.009	0.004		− 0.133	0.053	0.318		0.168	0.020	0.376
Indirect Effects													
*f*_0w_	→ DC_m_	→ Sat.DC_w_	− 0.042	0.001	0.314						0.012	0.001	0.792
*f*_0m_	→ DC_m_	→ Sat.DC_w_	0.083	0.003	0.072						0.010	0.001	0.840
*f*_0m_	→ DC_w_	→ Sat.DC_m_					0.059	0.003	0.080		− 0.021	0.001	0.504
*f*_0w_	→ DC_w_	→ Sat.DC_m_					− 0.025	0.001	0.463		0.071	0.002	0.110

*Note. N* = 187 mixed-gender couples; *f*_0_ = fundamental frequency; DC = coded dyadic coping behavior; Sat.DC = satisfaction with partner’s dyadic coping; w = women; m = men; β = standardized coefficients; SE = standard error. All significant coefficients are in bold (*p* < .05; two tailed).

*Group 1 (women stressed)*. When women were stressed, men’s *f*_0_ was significantly associated with men’s coping behavior (β = 0.25, *p* = .010) which in turn was associated with women’s satisfaction with their partner’s coping behavior (β = 0.33, *p* = .004), which supports the emotional resonance hypothesis. In addition to the hypothesized relationships, women’s level of satisfaction with their partner’s dyadic coping behavior was also influences by their own *f*_0_ (β = 0.23, *p* = .031) and partner’s *f*_0_ (β = 0.026, *p* = .019).

*Group 2 (men stressed)*. In contrast to group 1, evidence was found for the paraverbal stress communication hypothesis. Men’s *f*_0_ was significantly associated with women’s coping behavior (β = 0.23, *p* = .010) which in turn was associated with men’s satisfaction with their partner’s coping behavior (β = 0.026, *p* = .025).

**Fig. 2:**
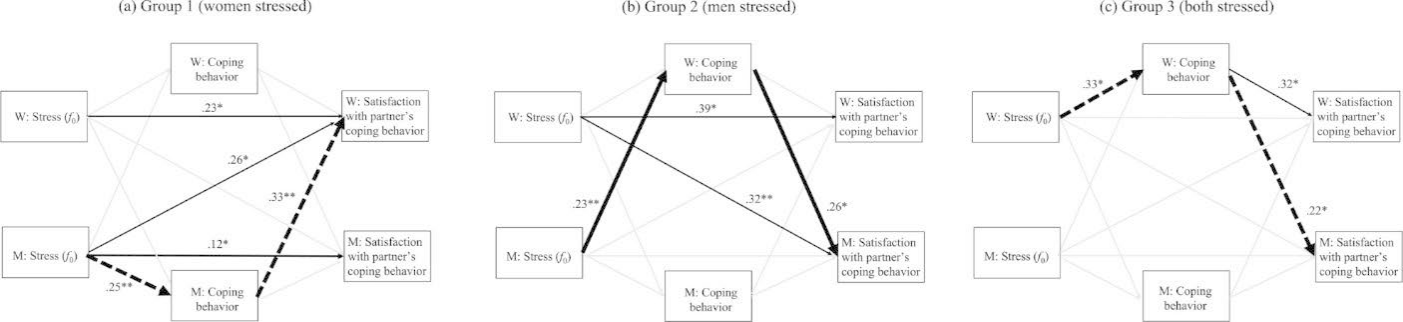
*Results for the Multi-Group APIMeM.* *Note*. In all three figures, bold black lines indicate confirmation of a hypothesis - a solid line represents the paraverbal stress communication hypothesis and a dashed line the emotional resonance hypothesis. It was found that if only the women were stressed (group 1), the emotional resonance hypothesis was confirmed; if only the men were stressed (group 2), the paraverbal stress communication hypothesis was supported; and if both partners were stressed, the emotional resonance hypothesis was validated. Thinner black lines show significant effects which were not theoretically predicted and gray lines show paths included in the model which are not significant. *p ≤ .05. **p ≤ .01. ***p ≤ .001.

*Group 3 (both stressed)*. Theoretically, it was unclear what happens when both partners are stressed. Our results show that women’s *f*_0_ affects their own coping behavior (β = 0.33, *p* = .030), which in turn influences men’s satisfaction with their partner’s support behavior (β = 0.22, *p* = .025), supporting the emotional resonance hypothesis for women but not for men. Beyond the hypothesized effects, we also found an actor-actor effect: Women’s own *f*_0_ affected their dyadic coping behavior, which in turn affected how satisfied they were with their partner’s coping behavior Fig. 2.

## Discussion

Dyadic coping enables couples to cope with stress originating from outside of the relationship by down-regulating one another’s stress-induced negative emotions and is, therefore, an important tool for romantic couples to enhance and maintain the well-being of their relationship (Bodenmann, 1995a, 2005). Yet, dyadic coping is a difficult process that requires the support-providing partner to correctly perceive and adequately respond to the support-seeking partner’s needs. The findings of the present study expand our knowledge by testing for a paraverbal stress communication hypothesis and an emotional resonance hypothesis. This allows us to differentiate whether the vocal stress of the stressed person matters to increase the partner’s dyadic coping behavior and whether the stress crosses over to the partner, facilitating more dyadic coping behavior.

### Paraverbal Stress Communication Hypothesis

In the condition when only men got stressed (Group 2), we found that men’s higher average *f*_0_ predicted more support provision by their partner provided, which in turn made those men more satisfied with the support they got. This is novel in two aspects. First, it shows that women do react to their partner’s voice and try to provide more support and, therefore, stress is communicated via one aspect of the voice, namely *f*_0_. Second, the process is based on a chain and not a mediation mechanism. *F*_0_ affects women’s support behavior, and the support behavior again influences men’s satisfaction with the provided support.

Prior studies on the same data set comparing Group 1 and 2 did not find gender differences. Those studies found that the cortisol level of the stressed partner reduced faster as a function of the support the non-stressed partner provided, particularly in men (Meuwly et al., 2012). Furthermore, more stress behaviors (i.e., asking the partner for advice, neutral, factual description, or emotion-focused expression of stress experience) are associated with more support provision by the partner (Bodenmann et al., 2015; Kuhn et al., 2017); and *f*_0_ is higher during verbal emotion-focused stress expressions (Bulling et al., 2020). Thus, gender effects seem to mostly occur when focusing on *f*_0_. Thus far, we can only speculate that either men are expressing stress more explicitly via *f*_0_ or that women are more sensitive to their partner’s para-verbal stress behaviors.

### Emotional Resonance Hypothesis

The emotional resonance hypothesis describes a potentially automatic process of emotional connection between partners by which the unstressed partner’s dyadic coping response might be improved (Leuchtmann et al., 2018). Evidence for the emotional resonance hypothesis was found in Group 1 and 3. In Group 1 (women stressed), men with higher *f*_0_ scores on average during the interaction provided more support to their partner, which supports the emotional resonance hypothesis. The findings could indicate that changes in the unstressed, support-providing partner’s voice—their paraverbal dyadic coping reactions—might be a prerequisite for feeling supported either by themselves or in combination with verbally (e.g., expressing compassion) or nonverbally (e.g., holding hands) expressed dyadic coping reactions. It is not clear if it was the unstressed partner’s voice alone that led to the stressed partner feeling of being more supported or if the stress resonance led to better verbal and nonverbal dyadic coping, as the partner was emotionally synchronized. If vocally expressed emotional arousal by the support provider is a prerequisite or facilitating factor for the stressed partner to feel more adequately emotionally supported. The question of whether and how this form of emotional resonance can be enhanced needs to be investigated. Nevertheless, *f*_0_ could be a communicative tool, because the stressed partner can now appraise by the partner’s voice that their message of stress experience has been well received and also affects the partner (sympathetic response). *f*_0_ is therefore not only related to more stress expression (Bulling et al., 2020), but also to more support. This might be linked to specific neural networks (inferior frontal gyrus, inferior parietal lobule) standing for emotional empathy (Shamay-Tsoory, 2011).

Thus, the present study extends findings from previous studies that found a positive association between dyadic coping and empathy (Levesque et al., 2014), clarity of emotion perception by the partner (mainly in men) (Leuchtmann et al., 2018), and in the similarity of partners’ ratings of their own emotions during dyadic interaction (Verhofstadt et al., 2008; Randall et al., 2021) found, emotional co-regulation is facilitated by positive dyadic coping and hindered by negative dyadic coping of both or higher negative dyadic coping of women (compared to men). Thus, it can be assumed that synchrony in *f*_0_ stands for positive dyadic coping (expression of understanding, empathy, show solidarity) and contradicts negative dyadic coping.

When both partners were stressed simultaneously (Group 3), the assumption of stress-coping behavior was less clear because both partners might compete to share their stressful experiences and to get support. The results support the emotional resonance hypothesis, as women’s higher *f*_0_ expression was linked to men’s higher support provision. However, it is less clear whether women with higher *f*_0_ were more empathic to their partner’s stress experience or if the higher level simply reflected their own stress experience. Thus, the interpretation in Group 3 is less straightforward, as own stress reaction and emotional responsiveness to the partner are intertwined.

In line with the findings for Group 1 and 2, we found that women’s dyadic coping influenced how satisfied their partners were with the provided support at the end of the interaction; however, we did not find that men’s support provision influenced women’s satisfaction level. Overall, there are several explanations for this less clear picture. First, it might be possible that the stress-coping process is undermined when both partners are stressed and the roles of providing and receiving support are mixed. Nevertheless, the satisfaction level with the received support was not different in comparison to stressed partners in Group 1 and 2. Second, the result could be an outcome of the specific research design. As both partners were stressed by the same stress induction (albeit apart), they found out quickly what had happened and solidarized. Thus, it might have been easy for both partners to empathize with the other, as they had experienced the same situation. This might be very different in cases when Partner A undergoes solely a stressful event and Partner B has a hard time empathizing as it is sometimes difficult to understand what the partner went through.

### Limitations and Future Research

The present study expands our understanding of the dyadic coping process. Using experimental stress induction and observing a couple interaction under natural, acute stress allows us to untangle whether the support-seeking and the support-providing partner’s emotional arousal may help or hinder the dyadic coping process depending on whether one or both partners are experiencing acute stress. Besides the experimental design, a strength of the study is that the variables were measured with different methods (i.e., automatically extracted *f*_0_, systematically coded dyadic coping behavior, self-reported satisfaction with the support the partner has provided), which reduces method bias (Podsakoff et al. 2012). Further, the dyadic framework of the data analysis allowed us to test for partner effects and thereby control for the interdependence between the romantic partners.

Still, the contribution of these findings should be evaluated in the context of several limitations of the sample and methodology. Our findings are limited in generalizability as couples in the study were young to middle-aged, mixed-gender, and primarily highly educated. Furthermore, we have been very explicit with the underlying causal assumptions yet the result can only be causality interpreted if the assumptions are correct (Pearl, 2009; Rohrer, 2018). Finally, using each participant’s mean *f*_0_ and summing up the observed dyadic coping behavior across the entire interaction is a simplified approach of testing the stress-coping process which naturally unfolds in real-time between the two partners. Thus, future studies might focus on a multi-modal approach to assess stress beyond just *f*_0_ (e.g., heart rate, electrodermal activity) as well as examine the stress-coping process on a more microscopic level. Results on multi-modal real-time processes might help the field to further develop theoretical assumptions about how the stress-coping process unfolds between partners.

Findings support the notion that strengthening dyadic coping in relationship education or couple therapy could benefit from the inclusion of paraverbal signals. Similar to biofeedback therapy, one could imagine developing a machine-learning-based tool that yields immediate feedback to partners on their *f*_0_ during dyadic coping processes. Combined with psychoeducation on cognitive and emotional empathy, providers or therapists could work on both dimensions, the emotional one (see three-phase-method; Bodenmann & Randall 2012), integrating emotional synchrony on the emotional and physiological level, and the cognitive one (Levy-Gigi & Shamay-Tsoory, 2017).

## Data Availability

Data and code can be accessed here: https://osf.io/ze6w8/files/osfstorage.

## Footnotes

1 Three prior articles (Bodenmann et al., 2015; Kuhn et al., 2017; Meuwly et al., 2012) have used this data set. However, this is the only one which focuses on f0, satisfaction with the provided support, and a comparison of the paraverbal stress communication and emotional resonance hypotheses.
